# REJECTION SENSITIVITY AS A MEDIATOR OF PERCEIVED SOCIAL ACCEPTANCE OF LGBTQ OLDER ADULTS

**DOI:** 10.1093/geroni/igz038.1107

**Published:** 2019-11-08

**Authors:** Kirenia Brunson, Sarah Nanami Morehouse, Ashely M Stripling

**Affiliations:** 1 Nova Southeastern University, Fort Lauderdale, Florida, United States; 2 Nova Southeastern University, Fort Lauderdale, United States

## Abstract

As the US population becomes older and more diverse, examinations exploring intersections of identity become increasingly important. Specifically, lesbian, gay, bisexual and transgender (LGBT) adults face unique challenges as they move into late life including issues related to caregiving, social isolation, and access to competent care. As such, understanding the role of moderating psychological factors, such as rejection sensitivity, within this population becomes instrumental to providing appropriate healthcare. Thus the purpose of this review is to (a) increase understanding of role of rejection sensitivity in LGBT older adults (b) understand the effect of treatment setting on this relationship. Source documents for this literature review were identified through PsycInfo, PsyARTCILES and Google Scholar using the following keywords, and combinations: “Older Adult,” “Gerontology,” “LGBTQ,” “gay,” “sexual minority,” “Rejection Sensitivity,” and “Social Acceptance”. Consistent with a thorough analysis, all English-Language abstracts were read, as were the full articles of those texts that appeared relevant to this literature review. After applying relevant exclusion criteria (i.e. young age), 6 texts warranted inclusion including: 2 books and 4 articles. A thorough review of the literature revealed that internalized homonegativity and rejection sensitivity mediate the degree of emotional dysregulation, internalization of symptoms and rejection-based proximal stress across multiple settings. However, the impact of these relationships in long-term care settings remains unknown. Implications of the current presentation include improving awareness of psychological health factors in LGBT older adults and provision of recommendations to reduce barriers to optimal care.

